# Myc is required for β-catenin-mediated mammary stem cell amplification and tumorigenesis

**DOI:** 10.1186/1476-4598-12-132

**Published:** 2013-10-30

**Authors:** Mejdi Moumen, Aurélie Chiche, Charles Decraene, Valérie Petit, Alberto Gandarillas, Marie-Ange Deugnier, Marina A Glukhova, Marisa M Faraldo

**Affiliations:** 1 Institut Curie, Centre de Recherche, F-75248 Paris, France; 2 CNRS, UMR144, F-75248 Paris, France; 3 Translational Research Department, Institut Curie, F-75248 Paris, France; 4 Fundación Marqués de Valdecilla-IFIMAV, 39008 Santander, Spain; 5 Inserm ADR Languedoc - Roussillon, F-34093 Montpellier, France

**Keywords:** β-catenin signaling, Mammary gland, Myc, Stem cells, Basal-like breast cancer

## Abstract

**Background:**

Basal-like breast cancer is a heterogeneous disease characterized by the expression of basal cell markers, no estrogen or progesterone receptor expression and a lack of HER2 ov erexpression. Recent studies have linked activation of the Wnt/β-catenin pathway, and its downstream target, Myc, to basal-like breast cancer. Transgenic mice K5ΔNβcat previously generated by our team present a constitutive activation of Wnt/β-catenin signaling in the basal myoepithelial cell layer, resulting in focal mammary hyperplasias that progress to invasive carcinomas. Mammary lesions developed by K5ΔNβcat mice consist essentially of basal epithelial cells that, in contrast to mammary myoepithelium, do not express smooth muscle markers.

**Methods:**

Microarray analysis was used to compare K5ΔNβcat mouse tumors to human breast tumors, mammary cancer cell lines and the tumors developed in other mouse models. Cre-Lox approach was employed to delete Myc from the mammary basal cell layer of K5ΔNβcat mice. Stem cell amplification in K5ΔNβcat mouse mammary epithelium was assessed with 3D-culture and transplantation assays.

**Results:**

Histological and microarray analyses of the mammary lesions of K5ΔNβcat females revealed their high similarity to a subset of basal-like human breast tumors with squamous differentiation. As in human basal-like carcinomas, the Myc pathway appeared to be activated in the mammary lesions of K5ΔNβcat mice. We found that a basal cell population with stem/progenitor characteristics was amplified in K5ΔNβcat mouse preneoplastic glands. Finally, the deletion of *Myc* from the mammary basal layer of K5ΔNβcat mice not only abolished the regenerative capacity of basal epithelial cells, but, in addition, completely prevented the tumorigenesis.

**Conclusions:**

These results strongly indicate that β-catenin-induced stem cell amplification and tumorigenesis rely ultimately on the Myc pathway activation and reinforce the hypothesis that basal stem/progenitor cells may be at the origin of a subset of basal-like breast tumors.

## Introduction

Microarray gene expression profiling of human primary breast tumors has identified five main cancer subtypes: luminal A and B, Her-2+, basal-like and normal-like [1-3]. In the last years, additional molecular subtypes have emerged including claudin-low and apocrine tumors [4,5]. The basal-like subtype, accounts for 15 to 20% of all breast cancers and is characterized by the expression of mammary basal cell markers, such as keratins 5 and 14 (K5 and K14 respectively) and the transcription factor p63. Basal-like tumors often exhibit poor differentiation and higher rates of proliferation and are frequently assimilated to triple-negative tumors, as neither express estrogen receptors (ER) nor progesterone receptors and both lack HER2 overexpression [1]. Metaplastic carcinomas, characterized by the differentiation of cancer cells towards squamous epithelium or mesenchymal elements, form part of the spectrum of basal-like tumors [6].

The transcription factor proto-oncogene Myc regulates the expression of many genes involved in the control of cellular metabolism, growth and proliferation [7]. Myc overexpression due to gene amplification or transcriptional regulation is commonly associated with human cancer. In breast cancer, Myc deregulation is associated with poor outcome [8] and recent studies have shown that Myc expression is particularly elevated in the basal-like (or triple-negative) subtype [9-11]. Myc is a transcriptional target of the Wnt/β-catenin, and activation of the Wnt/β-catenin signaling pathway has been linked to basal-like breast cancer [12,13].

The mammary epithelium consists of two layers: luminal secretory cells and basal myoepithelial cells. During lactation, luminal cells produce and secrete milk in response to hormone stimulation. In addition to the above mentioned basal epithelial markers, myoepithelial cells express smooth muscle (SM) contractile proteins, such as α-SM-actin and SM-myosin. Various studies in human and mouse models have suggested that multipotent stem cells capable of regenerating the mammary epithelium upon transplantation reside in the basal myoepithelial layer [14-16]. Unipotent lineage-restricted stem/progenitor cells have recently been identified by a lineage-tracing approach [17,18]. Although the mechanisms controlling the functions of mammary stem cells currently are understood only partly, several studies have pointed to Wnt/β-catenin pathway. First, Wnt factors have been shown to promote the self-renewal of mouse mammary epithelial cells capable of gland regeneration [19]. In addition, adult uni- and bipotent stem cells responsive to Wnt/β-catenin signaling have recently been described [17]. In human mammary epithelial cells, activation of the Wnt/β-catenin pathway after PTEN knockdown has been shown to induce the amplification of the stem/progenitor compartment [20].

It remains largely unknown how Wnt/β-catenin signaling promotes mammary stem cell expansion. A recent study has revealed that a Wnt target, the G-protein-coupled receptor Lrg5, is necessary for mammary stem cell activity [21]. We have shown that another Wnt/β-catenin target, Myc, is essential for mammary stem cell function in normal adult gland homeostasis [22]. However, it remains unknown, if Myc is required for mammary stem cell amplification and tumorigenesis driven by β-catenin, or, other effectors of Wnt/β-catenin signaling pathway can substitute for Myc in the control of mammary stem cell expansion and development of malignant breast lesions. Here, we address this question using a genetic approach. The transgenic K5ΔNβcat mice generated by our team display a constitutive activation of Wnt/β-catenin signaling due to the expression of a stabilized form of β-catenin in the mammary basal cell layer and develop mammary lesions resembling basal-like mammary carcinomas [23]. We show here that the stem cell pool is amplified in the preneoplastic glands of K5ΔNβcat mice. Furthermore, we found that Myc is required for this amplification and for β-catenin-induced tumorigenesis.

## Results

### Constitutive activation of β - catenin signaling in mammary basal cells induces triple-negative tumors

**Figure 1:**
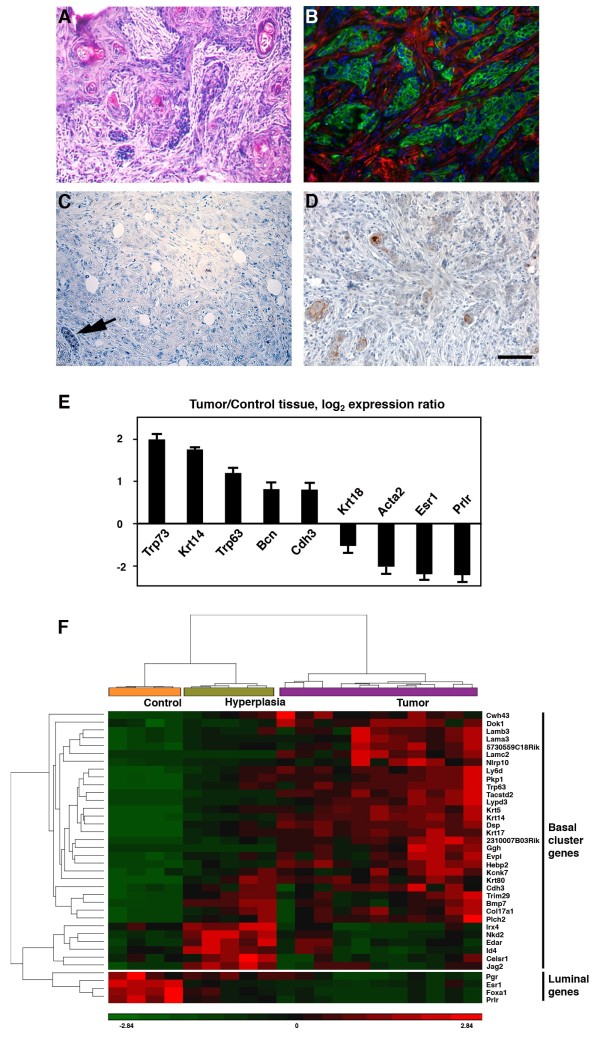
**K5ΔNβcat mammary tumors display characteristics of basal-like tumors. A**. Haematoxylin/eosin staining. **B**. Double immunofluorescence analysis with anti-K5 (green) and anti α-SMA (red) antibodies; nuclei are stained with DAPI. **C, D**. Immunohistochemistry with anti-ER **(C)** and anti-PR **(D)** antibodies. Arrow in C indicates the presence of normal ductal luminal cells positively stained for ER. Bar: 75 μm **(B,C)**, 150 μm **(A,D)**. **E**. qPCR analysis of representative basal and luminal genes. The graph shows mean values ± SEM for four control tissue and five K5ΔNβcat tumor samples; *p* < 0.05. **F**. Cluster analysis of K5ΔNβcat tumors, for the subset of genes defining the basal cluster in mouse tumor models (upper panel, [24]) and four luminal epithelial genes (lower panel). Data from 4 control mammary tissue, 5 hyperplasia and 11 tumor samples from K5ΔNβcat female are shown.

Female K5ΔNβcat mice develop mammary tumors with a median latency of 10 to 11 months [23]. Focal ductal hyperplasias, visible at early stages, progress to carcinomas, often presenting areas of squamous differentiation (Figure 1A). The tumors are highly proliferative and consist of cells positive for basal markers, including K5, K14 and p63, but negative for the smooth muscle proteins characteristic of myoepithelial cells, such as α-SMA and calponin (Figure 1B, Additional file 1: Figure S1A). These tumors stain negative for estrogen and progesterone receptors (ER and PR; Figure 1C,D). Moderate levels of ErbB2 were detected, but not higher than those found in normal tissue (Additional file 1: Figure S1B).

Affymetrix microarray gene expression analyses of K5ΔNβcat mouse mammary tumors and hyperplasias confirmed their basal characteristics, with an upregulation of basal genes and downregulation of luminal and smooth muscle genes. Quantitative RT-PCR (qPCR) analyses confirmed the upregulation of basal markers, such as *Krt14*, *Trp63*, *Trp73, Cdh3* and *Bcn,* and downregulation of the luminal genes *Krt18*, *Esr1*, *Prlr* and the smooth muscle marker, *Acta2*, with respect to control tissue (Figure 1E).

We performed a hierarchical clustering analysis of control and K5ΔNβcat mice datasets with a previously established basal gene signature [24]. We identified three distinct sample groups, corresponding to normal tissue, hyperplasia and tumors (Figure 1F). Most of the basal genes investigated were upregulated in the mammary tumors of K5ΔNβcat mice, whereas luminal genes were downregulated. Moreover, a combined hierarchical clustering analysis of our K5ΔNβcat dataset and datasets for human breast cancer cell lines [25] or basal breast tumors [26] showed a clustering of K5ΔNβcat tumors with the basal breast cancer lines and basal-like breast tumors (Additional file 2: Figure S2).

Altogether these results indicate that the constitutive activation of β-catenin signaling in the mammary basal cell layer induces tumors with gene expression profiles similar to that of basal-like human breast carcinomas and morphological similarities to malignant metaplastic human breast lesions.

### Myc is required for the formation of K5ΔNβcat tumors

**Figure 2:**
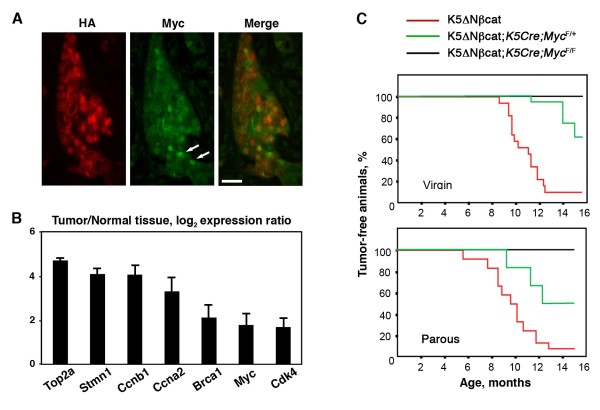
**Myc is necessary for mammary tumor formation in K5ΔNβcat animals. A**. Double immunofluorescence analysis of a K5ΔNβcat tumor with anti-HA (red), and anti-Myc (green) antibodies. Arrows indicate the presence of nuclei positively stained for Myc. Bar: 40 μm. **B**. qPCR analysis of Myc target genes. The graph shows mean ± SEM for four control tissue and five K5ΔNβcat tumor samples; *p* < 0.05. **C**. Kaplan-Meier tumor-free survival curve of K5ΔNβcat virgin and parous mice. 17 virgin and 10 parous K5ΔNβcat, 14 virgin and 6 parous K5ΔNβcat;*K5Cre;Myc*^*F/+ *^and 12 virgin and 10 parous K5ΔNβcat; *K5Cre;Myc*^*F/F *^females were analyzed.

Several previously identified β-catenin target genes, including *Myc,* were upregulated in K5ΔNβcat tumors, as shown by microarray and qPCR analyses (Figure 2B; Additional file 3: Figure S3A). Of note, the expression of Cyclin D1, one of the first identified β-catenin targets, was not altered in tumor tissue (Additional file 3: Figure S3A). Ingenuity pathway analysis (IPA) of microarray data showed that the Myc pathway was activated in K5ΔN βcat tumors (Additional file 3: Figure S3B). We found that Myc accumulated in the nuclei of tumor cells, often co-localizing with the HA-tagged transgenic protein (Figure 2A), and the upregulation of several Myc target genes in K5ΔN βcat tumors was confirmed by qPCR (Figure 2B).

To study whether Myc contributed to K5ΔN βcat mammary tumor formation, we conditionally deleted *Myc* gene from the basal cell layer of K5ΔN βcat mice by crossing them with mice carrying conditional alleles for *Myc* (*Myc*^*F/F*^) and *K5Cre* mice. Cohorts of K5ΔN βcat, K5ΔN βcat; *K5Cre; Myc*^*F/+*^ and K5ΔN βcat; *K5Cre;Myc*^*F/F*^ females were monitored for tumor formation until the age of 15 months (Figure 2C). In 90-95% of K5ΔN βcat females, palpable mammary tumors developed with a mean latency of 11 and 9.6 months for virgin and parous females, respectively. In contrast, only less than half of the females heterozygous for Myc in basal cells (K5ΔN βcat; *K5Cre; Myc*^*F/+*^) developed tumors (Figure 2C). Furthermore, tumor latency was delayed (14 and 11.2 months for virgin and parous females respectively), and the number of tumors per animal was reduced, when compared to K5ΔNβcat females (Table 1).

**Table 1:** **Tumor incidence in K5**Δ**Nβcat and K55**Δ**Nβcat;*K5Cre; Myc***^***F/W+***^**mice**

**Genotype**	**K5ΔN**β**cat**	**K5ΔN βcat; *K5Cre; Myc***^***F/+***^
Animals with tumor/total animals	25/29	8/20
Animals with 2 or more tumors	16	2

Animals were sacrificed at the age of 16 months.

**Figure 3:**
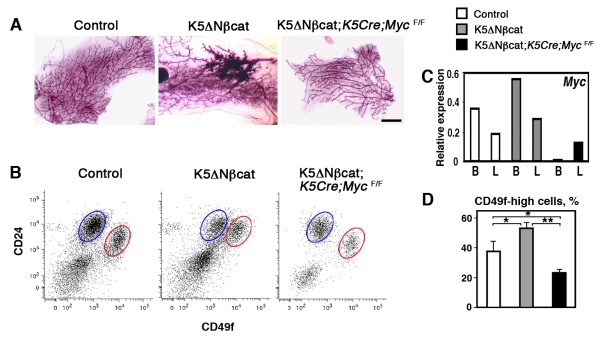
**Deregulated β-catenin signaling induces the expansion of the basal cell population. A**. Whole-mount carmine staining of mammary glands from 10-month-old mice. Bar: 3 mm. **B**. Separation of luminal (blue ovals, CD24^+^/CD49^low^) and basal (red ovals, CD24^+^/CD49^high^) epithelial cell populations from 10-month-old mice. **C**. qPCR analysis of *Myc* expression in freshly isolated basal (B) and luminal (L) mammary cell populations from 10-month-old virgin mice. **D**. Percentage of basal cells within the epithelial cell compartment (mean ± SEM of 3 independent experiments; *p < 0.05; **p < 0.001).

Strikingly, regardless of parity, none of the females with Myc-deficient basal cells (K5ΔN βcat; *K5Cre; Myc*^*F/F*^) developed palpable mammary tumors. Moreover, whole-mount mammary gland analysis revealed the absence of hyperplasia in K5ΔN βcat; *K5Cre; Myc*^*F/F*^ females (Figure 3A). Thus, Myc is required for mammary tumor formation in K5ΔNβcat mice.

### Stem/progenitor cell amplification induced by β-catenin signaling is mediated by Myc

As described previously, the mammary lesions observed in K5ΔNβcat females consist essentially of basal epithelial cells that lack myoepithelial differentiation [23]. We hypothesize that these lesions might result from an amplification of stem/progenitor cells in the mammary basal cell layer, due to Wnt/β-catenin signaling involving Myc activation. To investigate changes in stem cell activity in mutant mice, we isolated basal cells from 10-month-old virgin control, K5ΔNβcat and K5ΔN βcat; *K5Cre; Myc*^*F/F*^ females using flow cytometry cell sorting. Only K5ΔN βcat females with no palpable mammary tumors were chosen for these experiments. Whole-mount analysis revealed mild to moderate hyperplasia in most K5ΔN βcat mouse glands at this age (Figure 3A). Although the CD24 expression was up regulated in the basal cell population of K5ΔN βcat mouse glands, two epithelial cell populations — luminal (CD24^+^/ CD49f^low^) and basal (CD24^+^/CD49f^high^) — were efficiently separated (Figure 3B). We checked the purity of populations by qPCR analyses of K14 and K18 (basal and luminal keratins, respectively) in the sorted populations (Additional file 4: Figure S4). Analysis of gene expression confirmed the deletion of *Myc* from the basal cell population of K5ΔN βcat; *K5Cre; Myc*^*F/*^mice (Figure 3C).

Basal cells accounted for 37.8 ± 6% of the total mammary epithelial cell population in control mice and 53.4 ± 3.5% in K5ΔN βcat mice, indicating an amplification of the basal cell compartment induced by the activation of β-catenin signaling (Figure 3D). By contrast, in mutants presenting *Myc* deletion from the basal cell layer, K5ΔN βcat; *K5Cre; Myc*^*F/F*^ mice, the mammary basal compartment was smaller, accounting for only 23.4 ± 1.5% of the total mammary epithelial cells population (Figure 3D).

**Figure 4:**
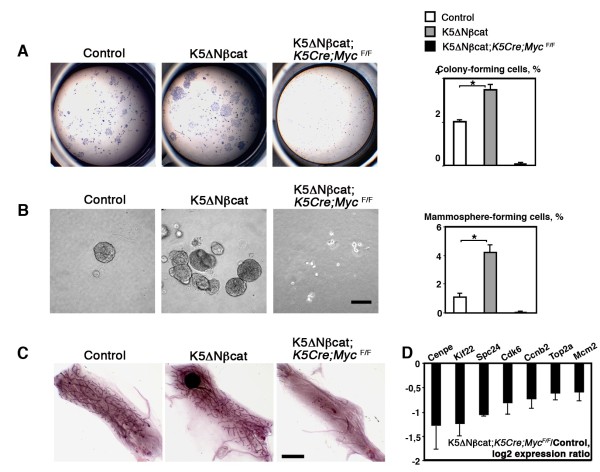
**Myc is required for stem cell amplification induced by deregulated β-catenin signaling. A**. Clonal colonies formed by 1000 sorted mammary basal cells. The graph shows the percentage of clonogenic cells (mean ± SEM of 3 independent experiments; *p < 0.01). **B**. Sphere formation by sorted basal cells. The graph shows the percentage of sphere-forming cells (mean ± SEM of 3 independent experiments; *p < 0.01). Bar: 100 μm. **C**. Mammary epithelial outgrowths developed from 1000 mammary basal cells transplanted into the cleared mammary fat pads. Bar: 4 mm. **D**. qPCR analysis of proliferation-related genes. Basal cell samples from K5ΔNβcat;*K5Cre;Myc*^*F/F *^and control mice obtained in 3 independent sorting experiments were analyzed.

To analyze the mammary stem/progenitor population, sorted basal cells were first tested in two-dimensional colony-formation assays [15]. The number of colony-forming cells was 1.8 times higher in K5ΔNβcat females than in controls (Figure 4A). The colonies displayed higher levels of cell proliferation, as determined by BrdU incorporation analysis, the percentage of BrdU-positive cells being 16.35 ± 0.4 for control cells and 23.6 ± 1.1 for K5ΔN βcat cells (*p* < 0.03; data from three independent cell sorting experiments). Basal cells isolated from K5ΔN βcat; *K5Cre; Myc*^*F/F*^ females were unable to form colonies (Figure 4A).

Sorted mammary epithelial cells have been reported to form mammospheres when cultured in the presence of 2% Matrigel, a property attributed to stem and progenitor cells [27]. As shown in Figure 4B, K5ΔN βcat basal cells formed four times more mammospheres than control cells, and the absence of Myc completely prevented mammosphere formation.

To further analyze the mammary stem/progenitor cell activity in mutant mice we transplanted sorted mammary basal cells at limiting dilutions (from 500 to 10 cells) into the cleared fat pad of prepubertal mice (Table 2). In all cases, basal cells from K5ΔN βcat glands produced normal mammary outgrowths with no tumor development (Figure 4C). The repopulating unit frequency of the basal population was 1/66 (1/134-1/32) for controls and 1/14 (1/23-1/8) for mutants (Table 2). Thus, the activation of β-catenin signaling increased the frequency of functional stem cells in the mammary basal cell layer by a factor of five. Basal cells from K5ΔNβcat;* K5Cre; Myc*^*F/F*^ mice generated no mammary outgrowths upon transplantation even, when 2000 cells were transplanted (Figure 4C).

**Table 2:** Limiting dilution transplantation of mammary basal cells from control and K5ΔN βcat mice

**Number of transplanted cells**	**Control**	**K5ΔN βcat**
500	3/3	3/3
100	3/4	4/4
20	3/12	10/12
10	3/11	5/11

Repopulating unit frequency in the basal cell population, as determined in limiting dilution transplantations: 1/66 (1/134-1/32) for control and 1/14 (1/23-1/8) for K5ΔNβcat; 95% CI, *p* value = 0.000284.

We recently showed that *Myc* deletion from the mammary basal cell layer affects stem cell activity [22]. Consistent with data for other tissues [7,28] microarray analysis of *Myc*-deficient mammary basal cells showed impaired expression of numerous genes involved in important cell functions, including metabolism, replication, protein synthesis and the cell cycle, relating to the capacity of the cell to proliferate (Additional file 5: Table S1). Many of these genes were deregulated in the mammary tumors developed by K5ΔNβcat mice (Additional file 6: Table S2). Interestingly, about half of these genes (40 out of 85) were found to be upregulated in the basal-like human breast tumors [26], (Additional file 6: Table S2). The expression of a set of proliferation-related genes upregulated in K5ΔNβcat tumors was analyzed by qPCR in mammary basal cells from K5ΔNβcat;*K5Cre;Myc*^*F/F*^ mice and control basal cells. In the absence of Myc, these genes were downregulated (Figure 4D), suggesting that *Myc* deletion impeded β-catenin-induced tumorigenesis, probably by restricting the acute growth program required for the amplification of basal progenitors and tumor formation.

## Discussion

We show here that constitutive stimulation of β-catenin signaling in the mammary basal cell layer of transgenic mice, led to development of triple-negative tumors that required activation of the Myc pathway. These tumors displayed transcriptional profile resembling that of human breast basal-like carcinomas and presented morphological similarities with metaplastic breast carcinomas, a basal-like tumor subtype [6].

Notably, K5ΔNβcat mammary tumors are different from those developing in MMTV-ΔN89βcat and MMTV-Wnt1 mice suggesting different cellular and molecular mechanisms underlying tumorigenesis. In MMTV-ΔN89βcat mice, luminal ER- tumors are thought to develop due to activation of β-catenin signaling in luminal progenitors, whereas in MMTV-Wnt1 model, “mixed” tumors containing luminal ER + and myoepithelial cells were suggested to originate from stem cells [29,30]. We show here that, as assessed in mammosphere and transplantation assays, in K5ΔNβcat mice, a basal stem/progenitor cell population is amplified at the early stages of malignancy, before the formation of basal-like invasive tumors with metaplastic characteristics.

The differences between breast tumor subgroups were hypothesized to reflect different types of mutation leading to tumorigenesis, or different cells of origin [31]. Two independent studies have indicated that basal-like Brca1-associated human and mouse mammary tumors originate from luminal epithelial progenitors [14,32]. The transformation of primary human breast basal-type epithelial cells has been reported to result in the development of metaplastic tumors [33]. Therefore, in light of our results, it is tempting to suggest that metaplastic basal-like breast tumors with squamous differentiation originate from basal progenitors or cells that acquired stem/progenitor properties due to enhanced β-catenin signaling. Of note, mammary lesions developed in K5ΔNβcat mice, consist of tumor cells that, in contrast to basal myoepithelial cells, do not express SM markers, suggesting that β-catenin activation either prevented SM differentiation of undifferentiated progenitors, or induced dedifferentiation of myoepithelial cell and acquisition of a progenitor phenotype, resulting in accumulation of basal cells with stem/progenitor properties. Transition between non-stem and stem-like states has been described in cultured human mammary basal epithelial cells [34]. Moreover, enhanced Wnt signaling in the intestinal epithelium has been found to induce dedifferentiation of non-stem cells and acquisition of tumor-initiating capacity [35]. Further studies involving the use of genetic markers of differentiated myopithelial cells are required to identify within the mammary basal compartment, the cell at the origin of K5ΔNβcat mouse tumors.

Genes encoding Myc and CyclinD1, two important Wnt targets, are often amplified and/or up-regulated in breast cancer [36]. Confirming previous analysis of preneoplasic glands [23], we have not found a significant up-regulation of Cyclin D1 in K5ΔNβcat tumors. This result is in agreement with previous studies reporting that Cyclin D1 is dispensable for Wnt or β-catenin induced tumorigenesis [37,38]. In K5ΔNβcat mammary tumors, similarly to previous findings in intestinal tumor models [39], Myc pathway appears activated and indispensable for β-catenin induced tumorigenesis. Interestingly, different studies have identified a Myc transcriptional gene signature to be associated with the basal-like breast cancer subtype [9-11]. In addition, Myc signaling has been shown to be upregulated in high-grade mammary tumors with presumptive cancer stem cell properties [40,41]. Therefore, Myc-targeting therapies are thought to be promising for specific treatment of different breast tumors [42]. The direct Myc inhibition seems difficult, and strategies used to target Myc include approaches focused on tumor cell metabolic abnormalities induced by Myc overexpression [43] or synthetic-lethal strategy [10,44]. A recent study has shown that cyclin-dependent kinase inhibition leads to regression of triple-negative tumors with Myc activation [10].

**Figure 5:**
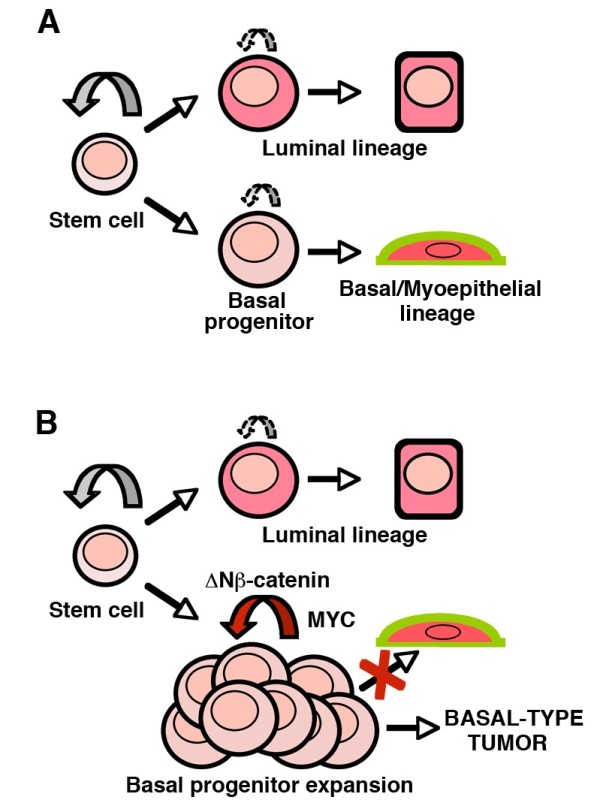
**Hypothetical scheme on the effects of β-catenin signaling activation on stem/progenitor population and tumor induction. A**. During normal homeostasis, basal and luminal progenitors with limited self-renewal potential, maintain their respective lineages. **B**. Constitutive activation of β-catenin signaling leads to enhanced basal progenitor self-renewal and expansion preventing myoepithelial differentiation and giving rise to basal-like tumors. Both, basal progenitor amplification and tumorigenesis are Myc-dependent.

We have recently shown that Myc is essential for mammary stem cell activity [22]. In agreement with previous reports [7], microarray analysis of Myc deficient basal cells shows an impaired expression of numerous genes involved in essential cell functions converging on the capacity of the cell to proliferate. An important part of these genes are upregulated in the K5ΔNβcat mammary tumors. Thus, our results indicate that Myc acts as the ultimate downstream effecter of β-catenin to provide the enhanced proliferative capacity to basal stem/progenitor cells leading to their amplification and tumorigenesis and preventing the myoepithelial differentiation (Figure 5). One important question to address in future studies is whether Myc deletion from established K5ΔNβcat tumors would lead to tumor regression. Such experiments would require inducible promoters permitting efficient gene deletion at desired time points.

In conclusion, our work provides new insights into the possible contribution of stem/progenitor cells to breast tumorigenesis and further confirms that the Myc pathway can be an interesting target for the development of basal-like breast cancer-tailored therapies.

## Methods

### Mice

K5ΔNβcat, *K5Cre* and *Myc*^*F/F*^ mice have been previously described [22,23,45-47]. All mice were bred in a 129SV/C57BL6 genetic background. Experiments were conducted in accordance with French veterinary guidelines and those of the Council of Europe for animal experimentation (L358-86/609EEC).

### Microarray analysis

Total RNA was extracted from mammary tissue with Trizol Reagent (Invitrogen), treated with DNAse and cleaned up on RNAeasy microcolumns (Qiagen). RNA was isolated from sorted basal cells in three independent biological pools of four to six control or mutant mice with the RNAeasy Microkit (Qiagen). Quality control was performed with an Agilent 2100 Bioanalyzer and the RNA 6000 Pico total RNA Kit (Agilent Technologies). As previously described [48], the WT-Ovation™ Pico RNA Amplification System and FL- Ovation™ cDNA Biotin Module V2 (Nugen) were applied to 1 ng of total RNA from sorted cells to generate biotinylated cDNA. Microarray analysis was carried out with Affymetrix GeneChip Mouse Genome 430 2.0 arrays. Expression data were normalized with the MicroArray Suite v.5.0 (MAS 5.0) algorithm and analyzed with the Partek Genomics Suite (Partek Incorporated). Differences in gene expression were considered to be validated whenever, for each ratio, the higher median gene expression value was up to 30 (corresponding to the estimated background cutoff, data not shown) and the corresponding fold-change was greater than 2. The microarray data have been deposited in the NCBI Gene Expression Omnibus and are accessible through GEO Series accession number GSE43825 (http://www.ncbi.nlm.nih.gov/geo/query/acc.cgi?token=rncxjwmcegeiuhw&acc=GSE43825).

### Clustering analysis

Hierarchical clustering analysis was carried out with Partek Genomics Suite Software (Partek, Inc.). The molecular and functional interactions of the genes identified were also analyzed with Ingenuity pathways analysis (IPA) tools (Ingenuity^®^ Systems) and a gene ontology approach (http://david.abcc.ncifcrf.gov). To compare the mouse and the human datasets, after GCRAMA normalization with Partek, a common correspondence between the mouse and human genes, was performed by using the annotation Tools package 2.35-8 in R 2.11.1 software and the HomoloGene database (ftp://ftp.ncbi.nlm.nih.gov/pub/HomoloGene).

### qPCR analysis

RNA was reverse-transcribed with MMLV H(-) Point reverse transcriptase (Promega), and quantitative PCR was performed by real-time monitoring of the increase in SYBR Green fluorescence on an ABI PRISM 7900HT Sequence Detection System (Applied Biosystems). The values obtained were normalized to *Gapdh* levels. The primers used for qPCR analysis are listed in Additional file 7: Table S3.

### Preparation of mammary epithelial cells and cell sorting analysis

The inguinal mammary glands of four to five females or hyperplastic mammary samples from two K5ΔNβcat females were pooled for the preparation of single-cell suspensions and cells were processed for flow cytometry as previously described [16,49]. The following conjugated antibodies were used: anti-CD24-PE (clone M1/69; BD Pharmingen), anti-CD49f-FITC (clone GoH3; BD Pharmingen), anti-CD45-APC (clone 30-F11; Biolegend), anti-CD31-APC (clone MEC13.3; Biolegend). Labeled cells were analyzed and sorted on a FACSVantage flow cytometer (Becton Dickinson, San Jose, CA, USA). Sorted cell populations were routinely reanalyzed and found to be 94 to 98% pure. Cell viability after sorting, as estimated by trypan blue exclusion, was between 80 and 90%.

### Transplantation assays

Sorted basal cells were resuspended in 10 μl of 50% growth factor-reduced Matrigel (BD Bioscience) and injected into the inguinal fat pads of three-week-old nude Balb/c females cleared of endogenous epithelium, as described elsewhere [49]. Mutant and control cells were grafted into contralateral fat pads of the same recipient mouse and outgrowths were analyzed 10 weeks after transplantation. Repopulating unit frequency was calculated with Extreme Limiting Dilution Analysis software [50].

### Cell culture assays

Sorted basal cells were cultured in DMEM/F12 medium containing 1% FCS and B27 supplement, at a density of 1000 cells per well [15]. For suspension sphere culture, sorted basal cells were plated on 24-well ultralow-attachment plates at 5000 cells/well and cultured in DMEM/F12 media containing B27, EGF, bFGF, heparin and 2% growth factor-reduced Matrigel (BD Bioscience, [27]. ImageJ software was used for quantification.

### Whole-mount analyses, histology and immunolabeling

Dissected mammary fat pads were spread onto glass slides, fixed in a 1/3/6 mixture of acetic acid/chloroform/methanol and stained with carmine (whole-mount staining). For histological analysis, samples were embedded in paraffin. Sections (7 μm thick) were cut and dewaxed for haematoxylin/eosin staining or immunolabeling, as previously described [23]. The following primary antibodies were used: mouse monoclonal anti-α-smooth muscle actin, anti-K14 (Sigma), anti-K8 (Covance) and anti-oestrogen receptor α (Dako); rat monoclonal anti-HA (Roche); rabbit monoclonal anti-calponin (Epitomics), polyclonal anti-K5, (Covance), anti-Ki67 (Novocastra), anti-c-erbB2, anti c-Myc and anti-PR (sc-284, sc-764 and sc-7208, respectively, Santa Cruz Biotechnologies). Alexafluor-conjugated secondary antibodies (1/1000, Molecular Probes) were used for immunofluorescence labelling. The Envision + System HRP kit (Dako) was used for immunohistochemistry.

### Statistical analysis of the data

All values are shown as mean ± standard error of the mean (SEM). P values were determined using Student’s test with two-tailed distribution and unequal variance.

## Competing interests

The authors declare that they have no competing interests.

## Authors’ contributions

MMF, MM, AC, MAD and VP performed experiments and analysed data, CD performed bioinformatic analyses of the microarray data, AG and MAD shared expertise, MAG and MMF designed the experimental plan and wrote the manuscript. All authors contributed to data analysis and interpretation and final approval of the manuscript. MMF and MAG contributed equally to this work.

## Supplementary Material

Additional file 1: Figure S1 Immunohistological analysis of K5ΔNβcat mammary tumors. A. Double immunofluorescence with antibodies against: K14 (red) and calponin (green) left panel; K5 (red) and K8 (green), central panel; HA (red) and Ki67 (green), right panel. B. Immunohistochemistry with the antibody against ErbB2 in normal mammary tissue (left) and a K5ΔNβcat hyperplasia (right). Bar: 75 μm (A), 150 μm (B). Click here for file

Additional file 2: Figure S2 Unsupervised hierarchical clustering of K5ΔNβcat tumor data with a dataset obtained from human breast cancer cell lines (A, ref. [25]) and breast tumors (B, Ref. [26]). Click here for file

Additional file 3: Figure S3 Gene expression analysis of K5ΔNβcat mammary tumors. A. Q-PCR analysis of Wnt/β-catenin pathway targets. The graph represent the mean values ± S.E.M. of four control tissue and five K5ΔNβcat tumor samples; p < 0.05. B. Ingenuity pathway analysis (IPA) of Myc pathway in K5ΔNβcat mammary tumors. The genes whose expression is increased in tumors with respect to normal mammary tissue are shown in red, and those whose expression is decreased, in green. Click here for file

Additional file 4: Figure S4 Q-PCR analysis of *Krt14*, and *Krt18* in freshly isolated basal (B) and luminal (L) cell populations from ten month-old virgin control, K5ΔNβcat and K5ΔNβcat; *K5Cre;Myc*^*F/F *^mouse mammary glands (the cell sorting experiment shown in Figure 3B). Click here for file

Additional file 5: Table S1 GOstat analysis of genes downregulated in *Myc*-deficient basal cells. Click here for file

Additional file 6: Table S2 GO analysis of the genes downregulated in Myc-deficient basal cells and upregulated in K5ΔNβcat hyperplasia. Click here for file

Additional file 7: Table S3 Primers used for qPCR analysis. Click here for file
