# Supplementary figures and images for: Myc is required for β-catenin-mediated mammary stem cell amplification and tumorigenesis
Source: Mol Cancer. 2013 Oct 30;12:132. doi: 10.1186/1476-4598-12-132 (PMC4176121; doi:10.1186/1476-4598-12-132)

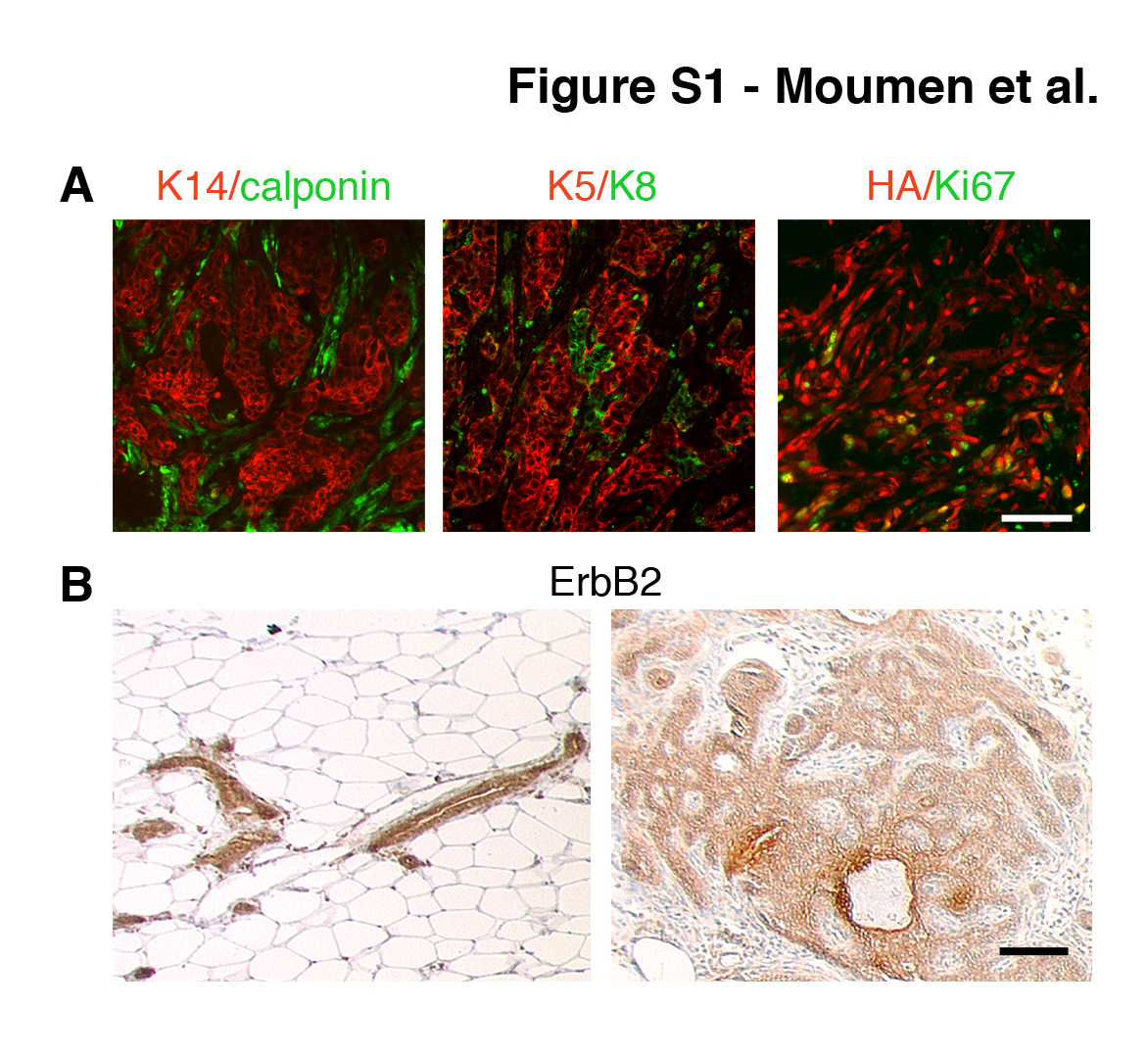

Supplement: Additional file 1: Figure S1 — Immunohistological analysis of K5ΔNβcat mammary tumors. A. Double immunofluorescence with antibodies against: K14 (red) and calponin (green) left panel; K5 (red) and K8 (green), central panel; HA (red) and Ki67 (green), right panel. B. Immunohistochemistry with the antibody against ErbB2 in normal mammary tissue (left) and a K5ΔNβcat hyperplasia (right). Bar: 75 μm (A), 150 μm (B). [file 1476-4598-12-132-S1.jpeg]

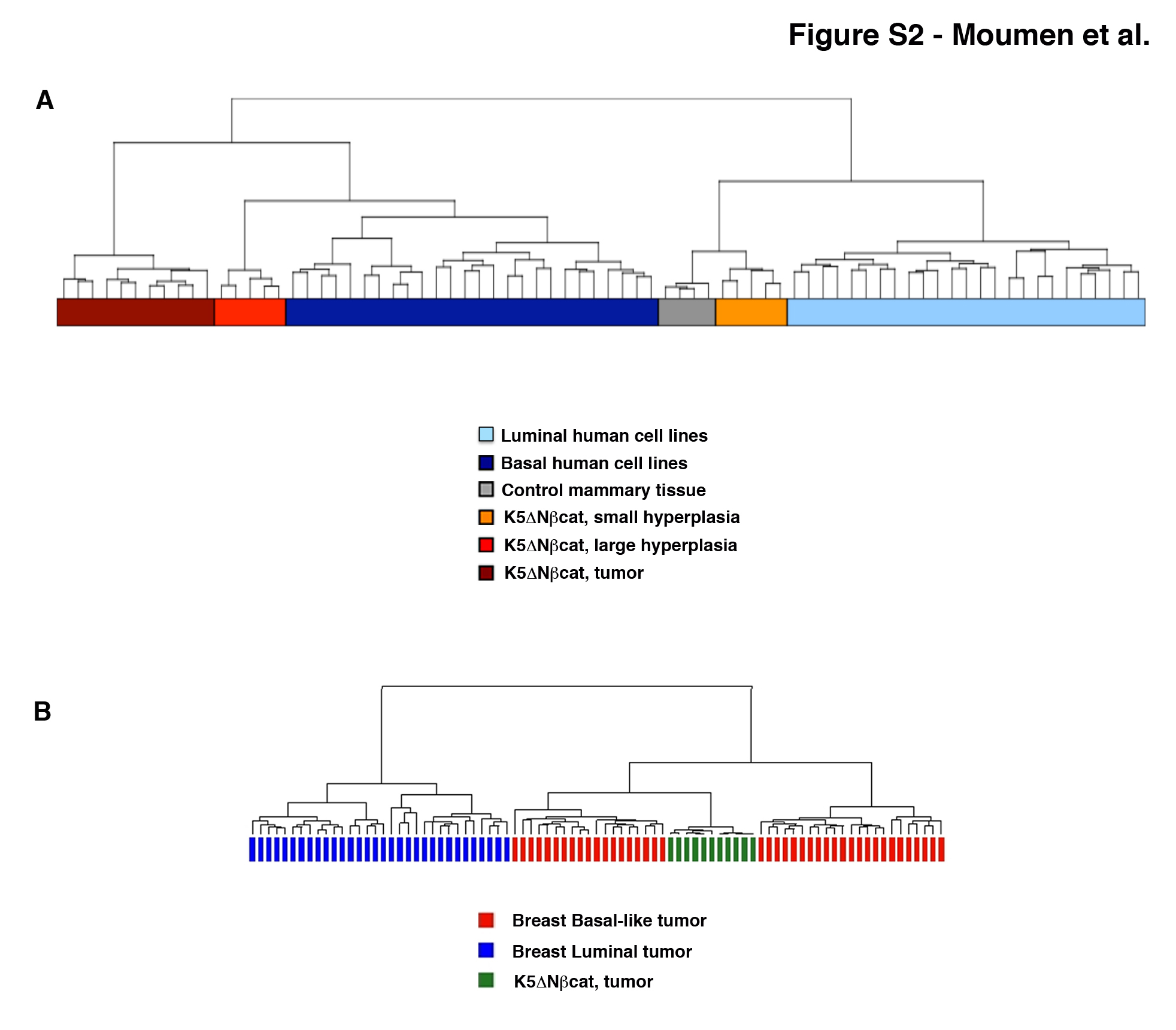

Supplement: Additional file 2: Figure S2 — Unsupervised hierarchical clustering of K5ΔNβcat tumor data with a dataset obtained from human breast cancer cell lines (A, ref. [25]) and breast tumors (B, Ref. [26]). [file 1476-4598-12-132-S2.jpeg]

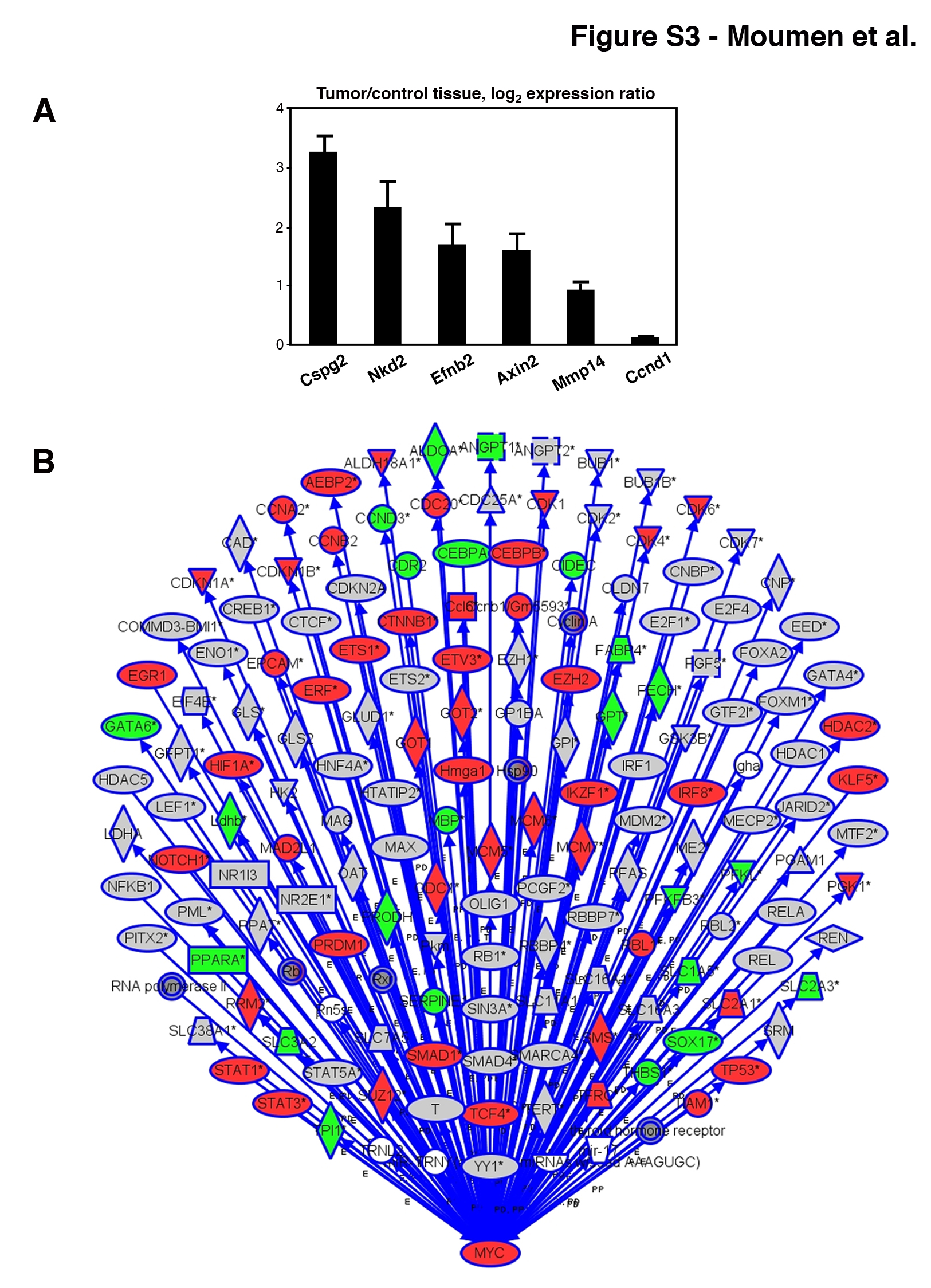

Supplement: Additional file 3: Figure S3 — Gene expression analysis of K5ΔNβcat mammary tumors. A. Q-PCR analysis of Wnt/β-catenin pathway targets. The graph represent the mean values ± S.E.M. of four control tissue and five K5ΔNβcat tumor samples; p < 0.05. B. Ingenuity pathway analysis (IPA) of Myc pathway in K5ΔNβcat mammary tumors. The genes whose expression is increased in tumors with respect to normal mammary tissue are shown in red, and those whose expression is decreased, in green. [file 1476-4598-12-132-S3.jpeg]

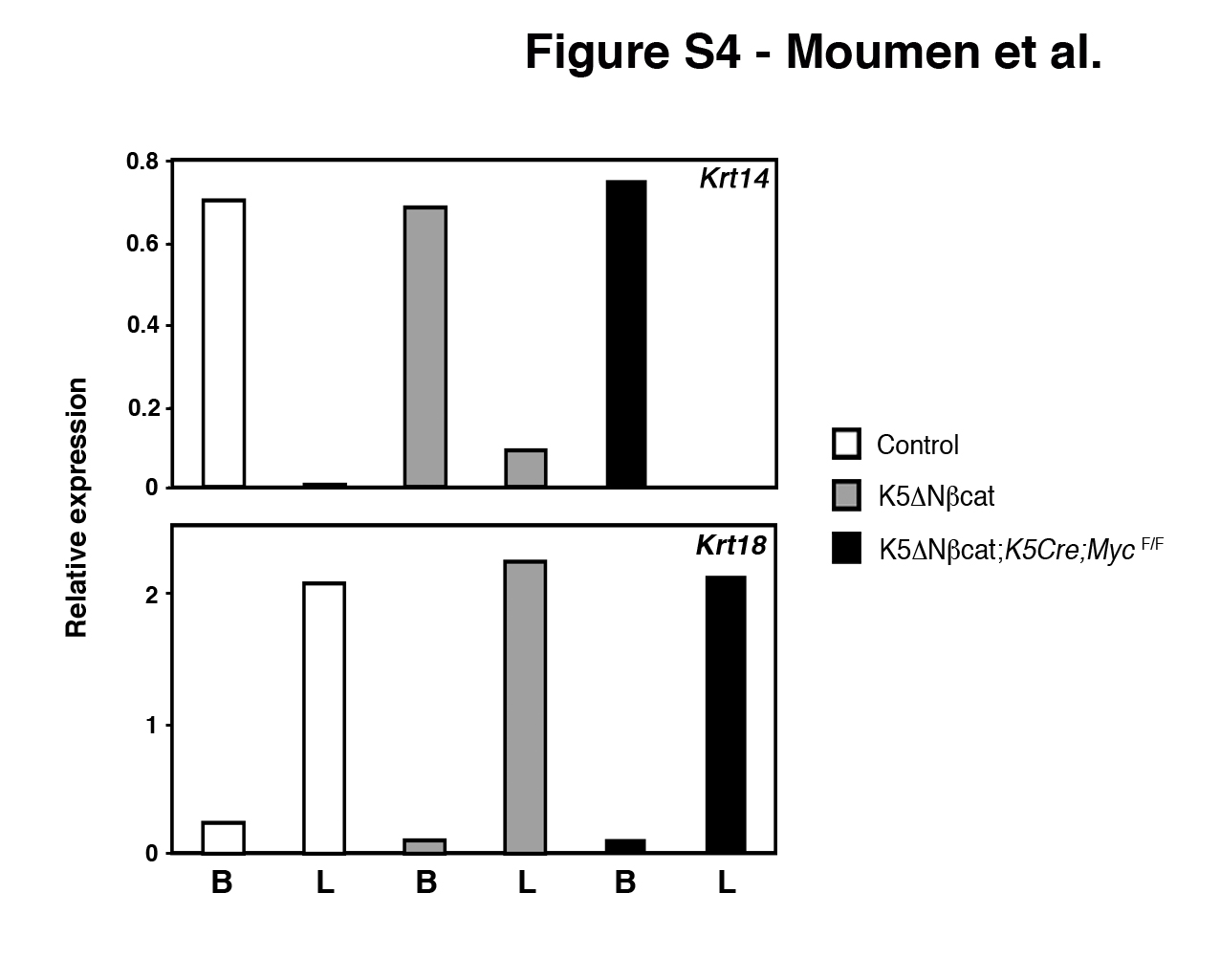

Supplement: Additional file 4: Figure S4 — Q-PCR analysis of Krt14, and Krt18 in freshly isolated basal (B) and luminal (L) cell populations from ten month-old virgin control, K5ΔNβcat and K5ΔNβcat; K5Cre;MycF/F mouse mammary glands (the cell sorting experiment shown in Figure 3B). [file 1476-4598-12-132-S4.jpeg]
