# Supplementary material for: Myc is required for β-catenin-mediated mammary stem cell amplification and tumorigenesis
Source: Mol Cancer. 2013 Oct 30;12:132. doi: 10.1186/1476-4598-12-132 (PMC4176121; doi:10.1186/1476-4598-12-132)
Supplement: Additional file 5: Table S1 — GOstat analysis of genes downregulated in Myc-deficient basal cells. [file 1476-4598-12-132-S5.doc]

**Table S1.** GOstat analysis of genes downregulated in *Myc*-deficient basal cells

|  | GO category | Number of genes (total)* | p-value |
| --- | --- | --- | --- |
| GO:0022613 | ribonucleoprotein complex biogenesis and assembly | 39 (174) | 1.15e-14 |
| GO:0044238 | Primary metabolic process | 472 (5694) | 4.23e-12 |
| GO:0006396 | RNA processing | 53 (330) | 1.09e-10 |
| GO:0006412 | translation | 57 (369) | 1.25e-10 |
| GO:0051301 | cell division | 41 (231) | 2.83e-10 |
| GO:0000278 | mitotic cell cycle | 39 (218) | 5.79e-10 |
| GO:0022403 | cell cycle phase | 44 (263) | 8.15e-10 |
| GO:0006399 | tRNA metabolic process | 23 (97) | 8.59e-10 |
| GO:0007154 | Cell communication | 82 (2387) | 2.66e-08 |
| GO:0006139 | nucleobase, nucleoside, nucleotide and nucleic acid metabolism | 244 (2709) | 2.66e-08 |
| GO:0010467 | gene expression | 223 (2452) | 7.91e-08 |
| GO:0007165 | Signal transduction | 76 (2178) | 3.67e-07 |
| GO:0006260 | DNA replication | 24 (129) | 1.82e-06 |
| GO:0009058 | biosynthetic process | 108 (1045) | 3.35e-06 |
| GO:0006996 | organelle organization and biogenesis | 98 (943) | 1.11e-05 |
| GO:0007166 | Cell surface receptor linked signal transduction | 33 (1170) | 2.38e-05 |
| GO:0043283 | biopolymer metabolic process | 303 (3757) | 2.83e-05 |
| GO:0006364 | rRNA processing | 14 (50) | 9.25e-05 |
| GO:0002376 | Immune system process | 9 (579) | 0.00012 |
| GO:0051276 | Chromosome organization and biogenesis | 37 (277) | 0.00012 |

*Number of affected genes in the ANOVA gene list, total number of genes in that category in parentheses.
