# Supplementary material for: Myc is required for β-catenin-mediated mammary stem cell amplification and tumorigenesis
Source: Mol Cancer. 2013 Oct 30;12:132. doi: 10.1186/1476-4598-12-132 (PMC4176121; doi:10.1186/1476-4598-12-132)
Supplement: Additional file 7: Table S3 — Primers used for qPCR analysis. [file 1476-4598-12-132-S7.doc]

**Table S3: Primers used for qPCR analysis**

Acta2-s 5’- GACCCAGATTATGTTTGAGAC - 3’

Acta2-as 5’- GTCCAGCACAATACCAGTTG - 3

Bcn-s 5’- CTATCGGCTGTACTCTGAAC - 3

Bcn-as 5’- CTATCGGCTGTACTCTGAAC - 3

Brca1-s 5’- CTGAGGGCATAAGAAACATTG - 3

Brca1-as 5’- GGCTCCACACACACATTTGA - 3

Ccnd1-s 5’- CATCAAGTGTGACCCGGACTG - 3

Ccnd1-as 5’- CCTCCTCCTCAGTGGCCTTG - 3

Cdh3-s 5’- acgaagcccctgtgtttgtt - 3

Cdh3-as 5’- gtcctgtgcggtatagatgc - 3

Cdk4-s 5’- CCTTCCCGTCAGCACAGTT - 3

Cdk4-as 5’- CCATCAGCCGTACAACATTG - 3

Cspg2-s 5’- ACCACGCTATGAAATCAACTC - 3

Cspg2-as 5’- GAAGGTGTCGCTGAATGAAAC - 3

Efnb2-s 5’- GTGAAGCCAAATCCAGGTTCT - 3

Efnb2-as 5’- CAGCACCACCAAAGTGATGA - 3

Esr1-s 5’- CTGGACAGGAATCAAGGTAAA - 3

Esr1-as 5’- GAGGCACACAAACTCTTCTC - 3

Gapdh-s 5’- CCAATGTGTCCGTCGTGGATC - 3’

Gapdh-as 5’- GTTGAAGTCGCAGGAGACAAC - 3’

Krt14-s 5’- CGGCAAGAGTGAGATTTCTG - 3’

Krt14-as 5’- TCCAGCAGGATTTTGTACTG - 3’

Krt18-s 5’- CCTTGCCGCCGATGACTTTA - 3’

Krt18-as 5’- CAGCCTTGTGATGTTGGTGT - 3’

Mmp14-s 5’- CTGGCGGGTGAGGAATAAC - 3’

Mmp14-as 5’- CAGGGAGGCTTCGTCAAACA - 3’

Myc-s 5’- CACCAGCAGCGACTCTGAA - 3’

Myc-as 5’- CCCGACTCCGACCTCTTG - 3’

Nkd2-s 5’- GTGCCCTACTGTGTGGATGA - 3’

Nkd2-as 5’- CAGGACCAAACTTAGATGTGT - 3’

Pgr-s 5’- CCACCTGTACTGCTTGAATAC - 3

Pgr-as 5’- CAACTGGGCAGCAATAACTTC - 3

Prlr-s 5’- ATAAAAGGATTTGATACTCATCTG - 3

Prlr-as 5’- GTCATCCACTTCCAAGAACTC - 3

Trp63-s 5’- TGCCCAGACTCAATTTAGTGA - 3’

Trp63-as 5’- GAGGAGCCGTTCTGAATCTG - 3’

Trp73-s 5’- CAGCCTTTGGTTGACTCCTA - 3

Trp73-as 5’- ACCGTGTACCTTGTTCATTG - 3

### Other primers were purchased from SABiosciences/Qiagen.
